# Metagenomes from 25 Low-Abundance Microbes in a Partial Nitritation Anammox Microbiome

**DOI:** 10.1128/mra.00212-22

**Published:** 2022-05-16

**Authors:** Natalie K. Beach, Kevin S. Myers, Timothy J. Donohue, Daniel R. Noguera

**Affiliations:** a Department of Civil and Environmental Engineering, University of Wisconsin-Madison, Madison, Wisconsin, USA; b Carollo Engineers, Inc., Broomfield, Colorado, USA; c Great Lakes Bioenergy Research Center, University of Wisconsin-Madison, Madison, Wisconsin, USA; d Wisconsin Energy Institute, University of Wisconsin-Madison, Madison, Wisconsin, USA; e Department of Bacteriology, University of Wisconsin-Madison, Madison, Wisconsin, USA; Indiana University, Bloomington

## Abstract

Microbial communities using anammox bacteria to remove nitrogen are increasingly important in wastewater treatment. We report on 25 metagenome-assembled genomes of low-abundance microbes from a partial nitritation anammox bioreactor system that have not been described previously. These data add to the body of information about this important wastewater treatment system.

## ANNOUNCEMENT

Processes utilizing anammox bacteria for nitrogen removal have become popular for energy-saving wastewater treatment since they do not require oxygen or organic carbon for growth to convert ammonium (NH_4_^+^) and nitrite (NO_2_^−^) directly to nitrogen gas (N_2_) (1–7). Microorganisms in partial nitritation anammox (PNA) bioreactors adapt to repetitive and rapidly changing microaerobic and anoxic environments (5). The most abundant and active functional groups involved in nitrogen cycling in PNA systems are anammox bacteria, ammonia-oxidizing bacteria, and nitrite-oxidizing bacteria, and some of the strains appear to be ubiquitous (8, 9); we are beginning to realize the extent of the metabolic versatility and interactions within these microbiomes (8–10). We reported on metagenome-assembled genomes (MAGs) of the most abundant and active microorganisms in a laboratory-scale PNA bioreactor treating reject water from a full-scale struvite recovery process (11). There, the bioreactor was inoculated with biomass from the York River Treatment Plant (Hampton Roads Sewerage District, Seaford, VA), which uses a PNA deammonification process to treat reject water from the solids dewatering facility (12).

This announcement includes 25 low-abundance MAGs with greater than 68% completion, grouped into 16 clusters, that were recovered from the same laboratory-scale PNA bioreactor and for which a specific role in the community has not been described (Table 1). These additional MAGs add to the expanding body of knowledge about microorganisms present in deammonification bioreactors at low abundance (e.g., each less than 1% based on DNA sequencing coverage and collectively less than 5%, determined as described previously [11, 13]).

**TABLE 1:** MAG statistics and genome accession numbers

MAG identification	GenBank accession no.	GTDB taxonomy	Completeness (%)	Contamination (%)	Genome size (bp)	No. of scaffolds	*N*_50_ (bp)	GC content (%)	Sequencing depth (×)	No. of tRNAs	No. of 5S rRNAs	No. of 16S rRNAs	No. of 23S rRNAs
BCT_39	JAJVIC000000000	*Bacteroidia* bacterium	99.52	3.02	4,874,625	55	472,994	42.53	87	45	2	2	2
BAC_79R	JAJVIK000000000	Bacterium	98.88	4.49	5,858,271	32	291,821	61.52	15	52	0	0	1
GAM_9R	JAJVIQ000000000	*Gammaproteobacteria* bacterium	97.62	2.41	3,557,069	39	193,302	66.55	32	49	1	0	0
BAC_63R	JAJVIF000000000	Bacterium	97.22	0	3,260,764	4	1,880,758	62.82	17	48	1	1	1
TMB_89R	JAJVIN000000000	*Thermoanaerobaculia* bacterium	97.01	0.85	6,170,929	69	148,422	65.82	16	48	1	1	1
BAC_38	JAJVIB000000000	Bacterium	95.54	1.1	7,631,068	45	347,644	58.95	126	48	1	1	1
ELM_99	JAJVIP000000000	*Elusimicrobia* bacterium	95.51	0	3,644,178	43	213,845	48.71	30	76	1	1	1
PCS_19R	JAJVHX000000000	*Phycisphaerae* bacterium	95.45	1.14	3,568,264	28	220,153	55.42	21	48	0	1	1
OMN_62	JAJVIE000000000	*Omnitrophota* bacterium	95.16	0	2,713,171	25	1,594,128	61.88	33	48	1	1	1
PCS_14R	JAJVHU000000000	*Phycisphaerae* bacterium	94.89	1.14	4,668,787	70	104,852	65.74	52	49	0	1	1
VRM_23R	JAJVHY000000000	*Verrucomicrobiae* bacterium	94.59	3.07	4,819,195	36	167,251	59.69	18	50	1	1	1
PCS_76R	JAJVII000000000	*Phycisphaerae* bacterium	94.32	0.57	5,487,514	72	107,025	67.82	16	54	1	1	1
PCS_15R	JAJVHV000000000	*Phycisphaerae* bacterium	93.18	1.7	5,249,578	50	159,786	67.13	59	61	0	1	1
ANL_81R	JAJVIM000000000	*Anaerolineae* bacterium	92.73	2.18	7,867,783	60	252,904	57.36	13	48	1	1	1
PCL_122_1R	JAJVHS000000000	*Phycisphaerales* bacterium	92.61	0.57	4,404,079	104	58,562	67.83	10	0	0	0	0
PSM_69	JAJVIG000000000	*Pseudomonadales* bacterium	91.16	0.86	3,834,276	66	84,489	68.46	29	46	0	0	0
GAM_33	JAJVHZ000000000	*Gammaproteobacteria* bacterium	89.29	3.48	4,271,281	128	64,536	64.03	45	52	1	0	0
MYX_71R	JAJVIH000000000	*Myxococcota* bacterium	89.03	0.65	3,968,878	78	77,580	63.12	11	44	1	1	0
RDC_77	JAJVIJ000000000	*Rhodocyclaceae* bacterium	88.03	0.21	2,815,254	67	57,548	64.68	18	39	0	0	0
PLM_90R	JAJVIO000000000	*Planctomycetota* bacterium	85.88	1.14	4,250,401	91	61,976	64.89	9	65	0	1	1
PYM_42R	JAJVID000000000	*Pyrinomonadales* bacterium	85.47	1.71	6,468,668	221	38,096	57.8	90	72	1	1	1
ANL_34	JAJVIA000000000	*Anaerolineae* bacterium	77.45	0.91	3,991,433	141	33,931	56.33	49	38	0	0	0
BAC_13	JAJVHT000000000	Bacterium	76.31	0	4,595,932	231	22,649	53.05	44	36	1	1	1
PLM_16R	JAJVHW000000000	*Planctomycetota* bacterium	75.57	2.84	3,864,190	174	25,696	72.1	42	51	1	1	1
BRK_7R	JAJVIL000000000	*Burkholderiaceae* bacterium	69.09	0.29	2,770,384	154	18,435	69.49	18	39	0	0	0

**FIG 1:**
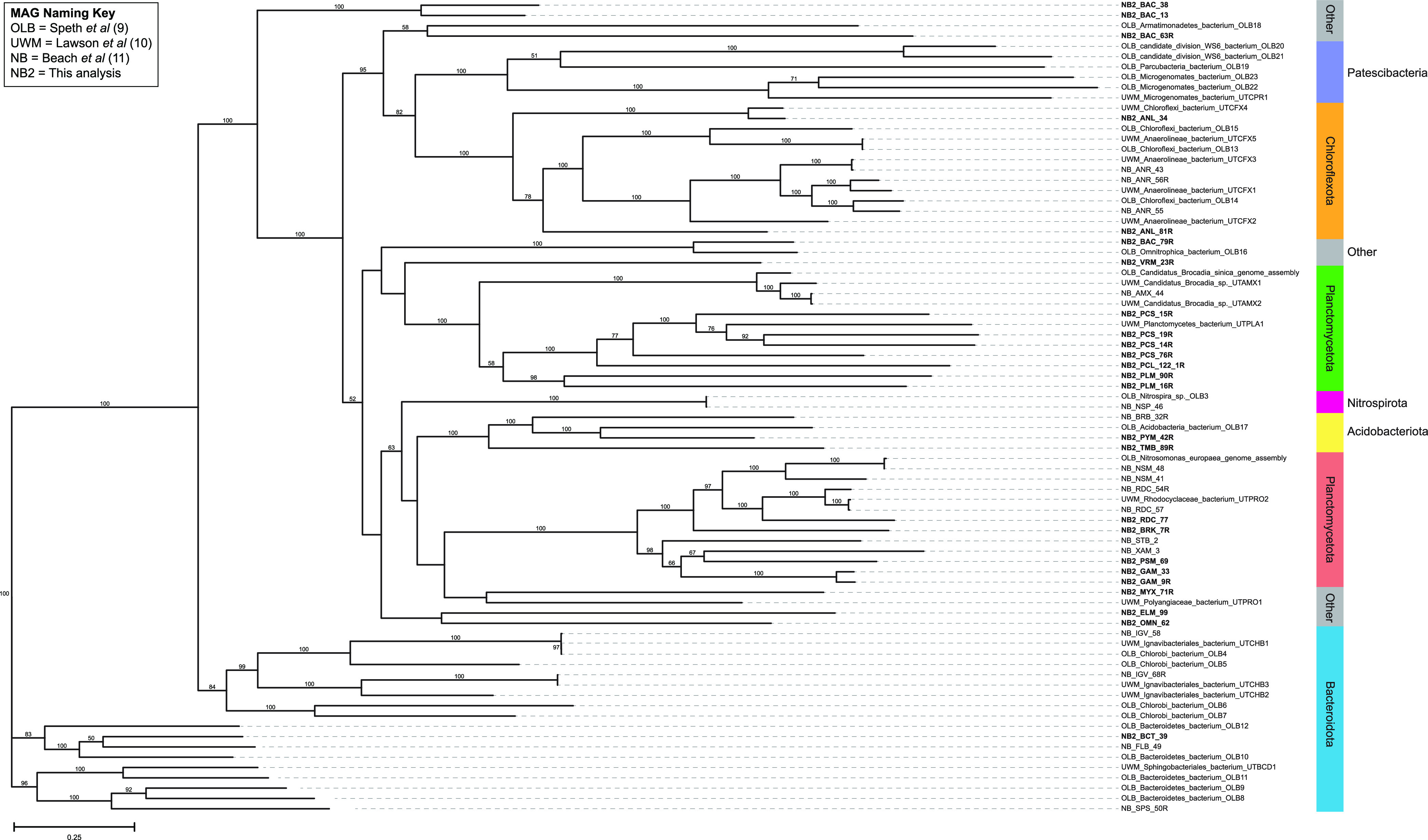
Phylogenetic tree of the 25 MAGs from this study (labeled NB2 and bolded), compared with the 16 most abundant MAGs from Beach et al. from the same microbiome (labeled NB) (11), as well as MAGs from Speth et al. (labeled OLB) (8) and Lawson et al. (labeled UWM) (9). The tree was constructed with RAxML-NG (unrooted) (25) with the 120 bacterial single-copy marker genes from GTDB-Tk (24) and visualized with TreeViewer (https://treeviewer.org). GTDB phylum taxonomy is listed on the right. Bootstrap values of more than 50 are shown. The scale bar indicates the number of nucleotide substitutions per sequence site. Strain codes for NB and NB2 strains are as follows: AMX, *Brocadia*; ANL, *Anaerolineae*; ANR, *Anaerolineales*; BAC, bacterium; BCT, *Bacteroidia*; BRB, *Bryobacteraceae*; BRK, *Burkholderiaceae*; ELM, *Elusimicrobia*; FLB, *Flavobacteriales*; GAM, *Gammaproteobacteria*; IGV, *Ignavibacteria*; MYX, *Myxococcota*; NSM, *Nitrosomonas*; NSP, *Nitrospira*; OMN, *Omnitrophota*; PCL, *Phycisphaerales*; PCS, *Phycisphaerae*; PLM, *Planctomycetota;* PSM, *Pseudomonadales;* PYM, *Pyrinomonadales*; RDC, *Rhodocyclaceae*; SPS, *Saprospiraceae*; STB, *Steroidobacteraceae*; TMB, *Thermoanaerobaculia*; VRM, *Verrucomicrobiae*; XAM, *Xanthomonadales*.

Genomic DNA was extracted at multiple time points during operation of the bioreactor (days 77, 231, 350, and 454) using a modified phenol-chloroform method (11). The quality of the isolated DNA was determined using a Qubit 4 fluorometer (Thermo Fisher Scientific, MA, USA), a NanoDrop 2000 spectrophotometer (Thermo Fisher Scientific), and gel electrophoresis. Sequencing libraries were prepared using the TruSeq DNA PCR-free kit (Illumina, CA, USA) following the standard protocol as described (14), samples were sequenced on the HiSeq 2500 platform (Illumina) at the University of Wisconsin-Madison Biotechnology Center using 2 × 250-bp reads, and low-quality reads (quality scores of less than 20 and sequence lengths of less than 100 bp) were removed using Sickle (15). A total of 18,106,239 reads from the metagenomic samples were processed with default parameters as described previously (11), using SPAdes v3.3.0 (16) for assembly, Anvi’o v5.5.0 (17) for binning, BBMap v38.22 (18) for mapping, Prodigal v2.6.3 (19), HMMER v3.2.1 (20), and NCBI Clusters of Orthologous Groups (COGs) (21) for annotation, and ProDeGe v2.2 (22) and tetranucleotide frequency comparisons for further cleaning of the MAGs. Genome statistics were determined with CheckM v1.0.3 (23), and taxonomy was assigned with GTDB-Tk v1.5.1 (database release 202) (24). The unrooted phylogenetic tree (Fig. 1) comparing these MAGs with MAGs from previous studies (8, 9) was generated using RAxML-NG v0.9.0 (25) and visualized with TreeViewer (https://treeviewer.org). These MAGs add to our understanding of PNA bioreactors and may aid in optimization of these systems once the function of these low-abundant microbes is elucidated.

### Data availability.

Raw metagenomic sequence data and MAGs are available in NCBI GenBank under BioProject accession number PRJNA559529. All custom scripts are available at GitHub (https://github.com/GLBRC/metagenome_analysis).
